# Contezolid in tuberculous meningitis: comparable efficacy and favorable safety

**DOI:** 10.3389/fcimb.2026.1955690

**Published:** 2026-09-08

**Authors:** Yanmei Liao, Fei Lv, Tianwen Quan, Li Zheng, Guihui Wu

**Affiliations:** 1 Department of Pharmacy, Public Health Clinical Center of Chengdu, Jinjiang District, Sichuan, Chengdu, China; 2 Department of Tuberculosis, Public Health Clinical Center of Chengdu, Jinjiang District, Sichuan, Chengdu, China

**Keywords:** contezolid, linezolid, real-world evidence, treatment outcomes, tuberculosis meningitis

## Abstract

**Background:**

Tuberculous meningitis (TBM) is the most severe and fatal form of tuberculosis (TB). Linezolid (Lzd), a key oxazolidinone with excellent central nervous system penetration, is widely used for TBM but is limited by significant adverse events (AEs). Contezolid (Czd), a next-generation oxazolidinone structurally optimized from Lzd, has shown promising potential. However, clinical evidence for its use in TBM remains limited to isolated case reports.

**Methods:**

We conducted a retrospective study of TBM patients treated with Czd- or Lzd-containing regimens. Patients were categorized into two groups: the Czd group (n=16, including 9 patients who switched from Lzd to Czd due to Lzd intolerance) and the Lzd group (n=32). We analyzed treatment outcomes, including Cerebrospinal fluid (CSF) parameters, imaging findings, and CSF culture conversion, as well as safety outcomes in both groups.

**Results:**

No AEs were observed in the Czd group, and hematological parameters remained stable. All Lzd-related AEs completely resolved after switching to Czd in the Lzd-to-Czd group. In the Lzd group, 68.75% experienced AEs, with 34.38% requiring dose reduction and 21.88% discontinuing treatment. Treatment outcomes were favorable in both groups: all 48 patients achieved clinical symptom improvement, with CSF Mtb culture conversion rates of 100% (Czd) vs. 93.33% (Lzd), and radiological improvement in 81.25% of patients in both groups. No significant differences were found in CSF parameter trends between the two groups (all P > 0.05).

**Conclusion:**

Czd represents an effective salvage strategy for Lzd intolerance and may prevent severe AEs while maintaining efficacy comparable to Lzd. These findings support Czd as a valuable alternative for TBM treatment.

## 1 Introduction

Tuberculosis (TB) is a global health issue that confers high morbidity and mortality. Tuberculous meningitis (TBM) is the most lethal form of TB, accounting for approximately 1% of all active TB cases and 5–10% of extrapulmonary TB cases worldwide (Fang et al., 2024). The disease burden is particularly severe in children under five years and in patients co-infected with HIV, where mortality can exceed 50% (Lourens et al., 2025; Çetin et al., 2025). Despite standard anti-TB treatment, mortality in adult patients with TBM reaches 30-60%, and more than 50% of survivors experience permanent neurological sequelae (Feng et al., 2021). This is largely attributable to the poor penetration of some drugs across the blood-brain barrier, which often results in suboptimal therapeutic concentrations in the cerebrospinal fluid and consequently limits treatment efficacy (Maranchick et al., 2022). Overall, delayed diagnosis, higher disease severity grade, and drug resistance have been consistently identified as key factors associated with unfavorable outcomes (Fang et al., 2024).

Linezolid (Lzd), a potent oxazolidinone antibiotic with excellent cerebrospinal fluid penetration, has emerged as a promising agent for TBM treatment (Fei et al., 2025). Observational studies have demonstrated that Lzd-containing regimens significantly improve clinical outcomes, particularly in critically ill patients, by reducing cerebrospinal fluid protein levels and enhancing survival (Fang et al., 2021). The World Health Organization has formally recommended Lzd for drug-resistant TB, and it serves as a cornerstone in modern regimens such as the BPaL regimen (Liu et al., 2024). However, the clinical utility of Lzd is severely constrained by its toxicity profile. Hematological toxicity occurs in up to 32.9% of patients, while peripheral neuropathy affects 35.6% during mid-to-late treatment stages (Chow et al., 2025), leading to premature treatment discontinuation in up to 40% of patients within three months (Abdelgawad et al., 2024). These adverse events (AEs) have become a critical clinical bottleneck, particularly for patients requiring prolonged therapy.

Contezolid (Czd) is a next-generation oxazolidinone antibiotic developed through structural optimization based on the chemical scaffold of Lzd. By replacing the morpholine ring with a 2,3-dihydropyridin-4-one (DHPO) ring, Czd retains comparable anti-TB activity while offering an improved safety profile, particularly concerning myelosuppression and monoamine oxidase inhibition (Xu et al., 2023). Studies have demonstrated its effective anti-TB activity in murine models (Almeida et al., 2023) and a significantly lower incidence of hematological AEs compared with Lzd in Phase III trials (Xu et al., 2023). However, clinical evidence for Czd in TBM remains extremely limited. To date, only a few case reports have documented its use in patients with TBM who were intolerant to Lzd. All reported favorable outcomes with resolution of Lzd-related AEs after switching to Czd (Guo et al., 2023; Liu et al., 2025; Wang et al., 2026; Wang et al., 2023; Xu et al., 2023). Pharmacokinetic data have demonstrated that Czd can penetrate the blood-brain barrier, with CSF concentrations exceeding the minimum inhibitory concentration (MIC) for Mycobacterium TB (Guo et al., 2023). Nevertheless, further studies are urgently needed to confirm its efficacy and safety in this devastating disease.

This study aimed to conduct a retrospective analysis comparing the efficacy and safety of Czd versus Lzd in the treatment of TBM, and to evaluate the clinical outcomes of patients who switched from Lzd to Czd due to intolerance, thereby providing real-world evidence to inform clinical decision-making.

## 2 Materials and methods

### 2.1 Study design and patients

**Figure 1:**
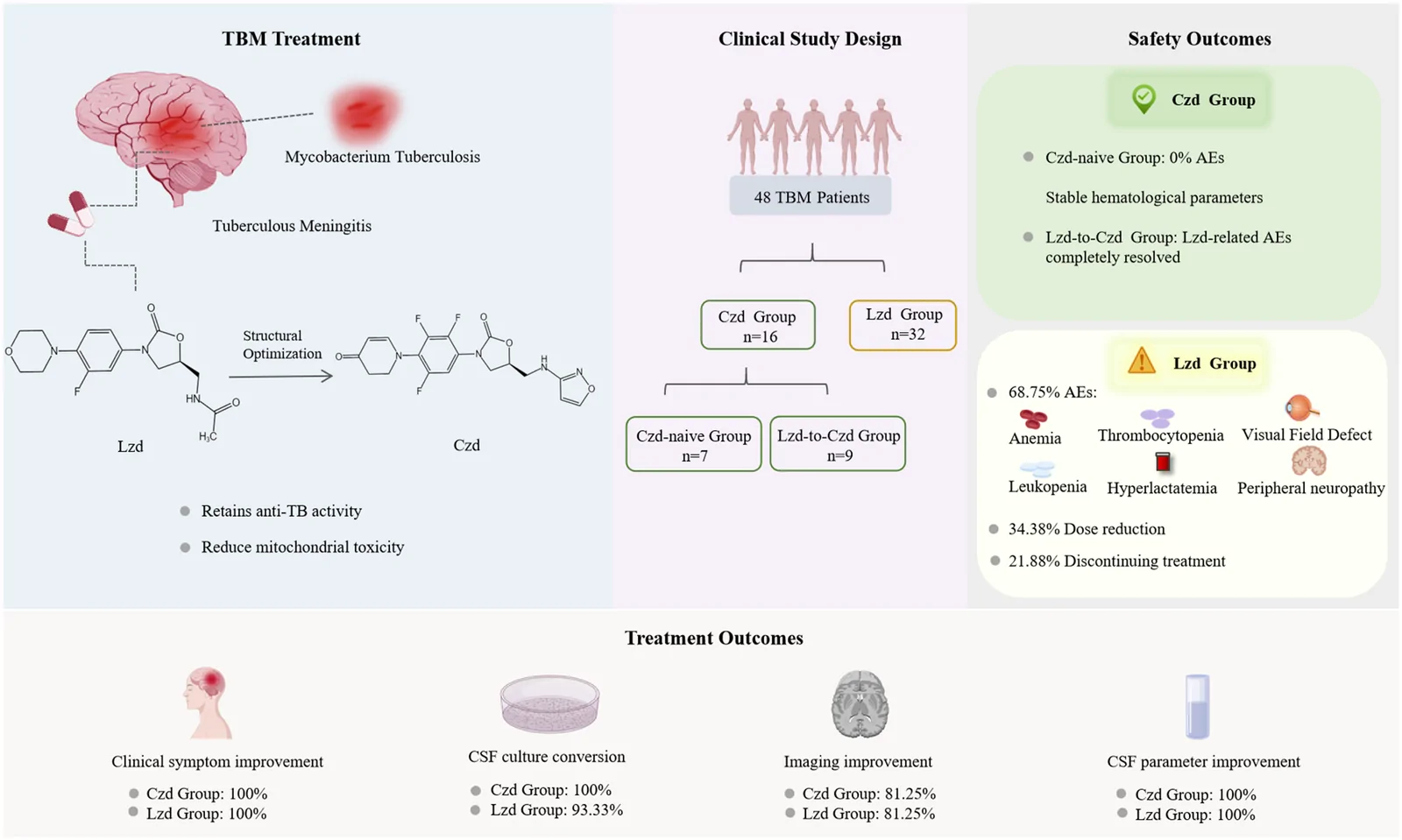
Clinical efficacy and safety of contezolid in the treatment of tuberculous meningitis. TBM: Tuberculous meningitis; Czd: Contezolid; Lzd: Linezolid; AEs: Adverse events; CSF: Cerebrospinal fluid. The elements in this figure were created using BioGDP.com (https://BioGDP.com)[15].

This single-center, retrospective, real-world study was conducted at the Chengdu Public Health Clinical Center and analyzed the medical records of 48 patients diagnosed with TBM between March 2022 and March 2026 (**Figure 1**) (Jiang et al., 2025). All eligible cases were identified through a search of the hospital information system and enrolled according to predefined TBM diagnostic criteria. The 48 patients were divided into two groups: the Czd group (n = 16), which included patients who were switched from Lzd to Czd due to intolerance (n = 9), and the Lzd group (n = 32). The primary objective was to evaluate the clinical efficacy and safety of Czd in the treatment of TBM, with particular emphasis on CSF parameters, improvement in intracranial lesions, overall treatment outcomes, and AEs. Data extracted from electronic medical records encompassed demographic characteristics, TB history, disease-related symptoms and signs (cough, sputum production, fever, headache, seizures, altered consciousness, etc.), TBM diagnostic classification, BMRC staging, detailed anti-TB treatment regimens (including drug composition, dosage, and duration), laboratory test results, AEs, and treatment outcomes (including CSF parameters and imaging examinations). Patients with HIV infection, incomplete laboratory data, or treatment duration of less than 2 months were excluded.

This study was conducted in accordance with the Declaration of Helsinki. Given the retrospective design, no direct patient intervention was performed during the study period. The protocol was reviewed and approved by the Ethics Committee of Chengdu Public Health Clinical Center (Approval No.: YJ-K2024-07-01). As this was a retrospective chart review without direct patient intervention, the requirement for informed consent was waived by the ethics committee, and all patient data were anonymized prior to analysis.

### 2.2 Treatment regimens

All patients received individualized anti-TBM regimens based on a comprehensive assessment of their clinical condition, treatment history, and available drug susceptibility profiles. Czd was administered orally at a dose of either 800 mg or 400 mg twice daily. The 800 mg q12h dose was chosen for patients with adequate baseline hematological parameters, while the 400 mg q12h dose was reserved for those with poor baseline hematological status (concurrent reductions in white blood cells, hemoglobin, and platelets), elderly patients, or those at high risk of myelosuppression, aiming to minimize bone marrow suppression while maintaining efficacy. This dosing strategy is consistent with the Chinese Expert Consensus on Contezolid for the Treatment of Tuberculosis (2025) and the pharmacokinetic profile of Czd. Lzd was administered orally at a dose of either 600 mg or 300 mg once daily. The selected oxazolidinone was combined with a tailored background regimen of 3 to 4 other anti-TB drugs, which included options such as isoniazid (H), rifampicin (R), ethambutol (E), pyrazinamide (Z), moxifloxacin (Mfx), levofloxacin (Lfx), clofazimine (Cfz), cycloserine (Cs), prothionamide (Pto) and amikacin (Am).

Background anti-TB agents were selected based on the following principles: (1) drug susceptibility testing results; (2) baseline liver function and renal function; (3) potential drug-drug interactions; (4) prior history of drug intolerance; and (5) the need for optimal central nervous system penetration in severe TBM cases. All regimens were individualized through multidisciplinary discussion, and treatment decisions were documented in the medical records.

### 2.3 TBM category and grading standards

TBM was classified into “Definite,” “Probable,” and “Possible” categories based on clinical standards, relevant CSF indicators, brain imaging criteria, and other TB related evidence (Marais et al., 2010).

Definite TBM: Diagnosis was confirmed by the presence of Mycobacterium tuberculosis in CSF via methods such as acid-fast bacilli staining, culture, or commercial nucleic acid amplification tests.

Probable TBM: This diagnosis was made if the total score from the diagnostic criteria was ≥ 10 without neuroimaging or ≥ 12 with neuroimaging.

Possible TBM: Diagnosis was considered when the total score was between 6 and 9 without neuroimaging or 6–11 with neuroimaging.

The severity of TBM at admission was assessed using the British Medical Research Council (BMRC) grading system, which is based on Glasgow Coma Scale (GCS) scores and focal neurological deficits (Tunkel, 2005):

Grade I: GCS score of 15 with no focal neurological deficits.
Grade II: GCS score of 15 with focal neurological deficits or GCS score of 11–14.
Grade III: GCS score ≤ 10.

### 2.4 Statistical analyses

**Figure 2:**
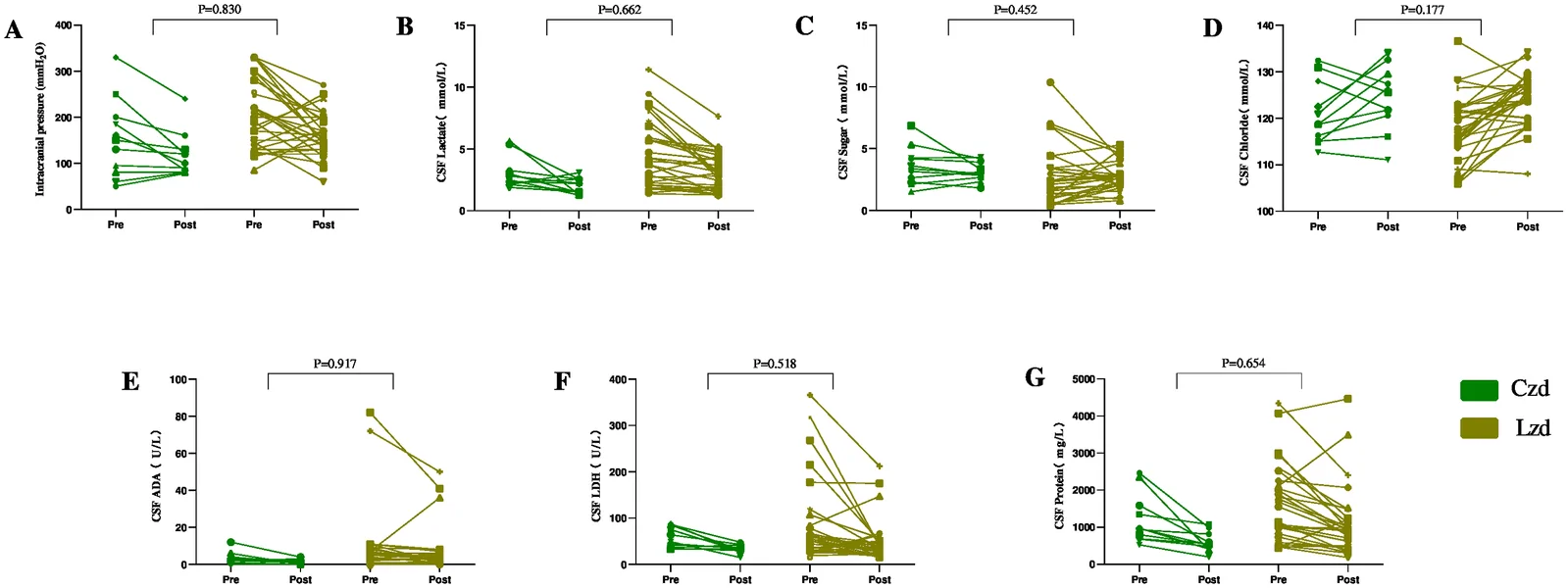
Changes in CSF parameters after two months of treatment. Panel A shows changes in intracranial pressure, while Panel B depicts changes in CSF lactate levels. Panels C and D illustrate changes in CSF glucose and chloride levels, respectively. Panels E and F highlight changes in CSF adenosine deaminase (ADA) and lactate dehydrogenase (LDH) levels, respectively. Panel G demonstrates changes in CSF protein levels in all patients. *P < 0.05. Of note, 7 patients in the Czd group and 2 patients in the Lzd group had normal baseline CSF parameters and did not undergo follow-up testing.

Statistical analyses were performed using IBM SPSS Statistics version 27.0 (IBM Corp., Armonk, NY, USA) and GraphPad Prism version 8.0 (GraphPad Software, San Diego, CA, USA). For comparisons between the Czd and Lzd groups, continuous variables with non-normal distribution were expressed as medians with interquartile ranges (IQR) and compared using the Mann–Whitney U test, while categorical variables were summarized as frequencies and percentages and compared using the Chi-square test or Fisher’s exact test, as appropriate. For within-group paired comparisons in the switch group, the Wilcoxon signed-rank test and repeated-measures one-way ANOVA were employed. For the comparison of longitudinal trajectories between the Czd and Lzd groups, repeated-measures two-way ANOVA was performed to analyze the time × group interaction effect. Individual trajectory plots were generated using GraphPad Prism to visualize the dynamic changes in hematological and CSF parameters. For longitudinal comparisons of CSF parameters (**Figure 2**), only patients with paired data at both baseline and follow-up were included (complete-case analysis). No imputation methods were applied for missing values, as the missingness was primarily due to normal baseline findings that did not warrant a repeat lumbar puncture. All statistical tests were two-sided, and a P value < 0.05 was considered statistically significant.

## 3 Results

### 3.1 Baseline characteristics

Based on the exclusion criteria, a total of 48 TBM patients were retrospectively included. Among them, 43.75% were female, and the median age was 42.50 (11.00–92.00) years. Mixed intracranial infection was present in 6.25% of patients. Headache (60.42%), fever (60.42%), and cough (60.42%) were the most common symptoms. Two patients (4.17%) experienced seizure episodes during the course of the disease. All 48 patients presented with extracranial TB and received glucocorticoids for anti-inflammatory therapy or mannitol for intracranial pressure reduction during treatment. According to the BMRC staging criteria, 32 patients were classified as stage II, 15 as stage I, and 1 as stage III (**Table 1**). Based on the Marais criteria, 21 patients were diagnosed with definite TBM and 27 with probable TBM. Of the 48 patients, 16 received Czd-containing regimens, while 32 received Lzd-containing regimens. The two groups were comparable in terms of age, sex distribution, baseline immune status, and clinical symptoms. No significant differences were observed between the groups regarding drug resistance profiles, background regimen composition, TBM grade and staging, and the severity of initial intracranial lesions on MRI abnormalities (**Table 1**).

**Table 1:** The demographic and clinical characteristics of the participants.

Characteristics	All participants (n=48)	CZD group (n=16)	LZD group (n=32)	P-value
Age, median years (IQR)	42.50 (11.00-92.00)	59.50 (25.00-92.00)	45.00 (11.00-81.00)	0.102
Female (%)	21 (43.75)	10 (62.5)	11 (34.38)	0.064
Mixed intracranial infection (%)	3 (6.25)	0	3 (9.38)	0.541
Seizures (%)	2 (4.17)	1 (6.25)	1 (3.13)	1
Extracranial TB (%)	48 (100)	16 (100)	32 (100)	1
Corticosteroid use (%)	48 (100)	16 (100)	32 (100)	1
CD4/CD8 < 1	9 (18.75)	3 (18.75)	6 (18.75)	1
Resistance category				0.4
Drug-susceptible (%)	41 (85.42)	15 (93.75)	26 (81.25)	
Isoniazid-resistance (%)	2 (4.17)	1 (6.25)	1 (3.13)	
Rifampicin-resistant (%)	1 (2.08)	0	1 (3.13)	
Multidrug-resistant (%)	4 (8.33)	0	4 (12.49)	
Use of background anti-TB drugs				
Isoniazid	35	12	23	1
Rifampicin	20	5	15	0.363
Ethambutol	17	7	10	0.524
Pyrazinamide	24	5	19	0.125
Levofloxacin	28	9	19	1
Moxifloxacin	15	5	10	1
Cycloserine	12	3	9	0.725
Prothionamide	11	1	0	0.333
Amikacin	11	1	10	0.073
Clofazimine	3	1	2	1
Symptoms				
Headache (%)	29 (60.42)	7 (43.75)	22 (68.75)	0.095
Fever (%)	29 (60.42)	14 (87.5)	15 (46.88)	0.007
Cough (%)	29 (60.42)	9 (56.25)	20 (62.5)	0.676
Nausea and vomiting (%)	15 (31.25)	0	15 (46.88)	<0.001
Consciousness disturbance (%)	17 (35.42)	10 (62.5)	7 (21.88)	0.006
Diagnostic category				0.537
Definite TBM (%)	21 (43.75)	6 (37.5)	15 (46.88)	
Probable TBM (%)	27 (56.25)	10 (62.5)	17 (53.12)	
BMRC staging				0.122
I (%)	15 (31.25)	3 (18.75)	12 (37.5)	
II (%)	32 (66.67)	12 (75.00)	20 (62.5)	
III (%)	1 (2.08)	1 (6.25)	0	
MRI abnormalities				
Infarction (%)	14	7	7	0.116
Meningeal enhancement (%)	24	6	18	0.221
Ventricular enlargement (%)	17	7	10	0.393
Hydrocephalus (%)	5	2	3	1

### 3.2 Treatment outcomes

Treatment outcomes were favorable in both groups. All 48 patients achieved clinical symptom improvement (**Table 2**). In the Czd group, all 6 patients with definite TBM had positive CSF cultures converted to negative, while the remaining 10 patients with probable TBM maintained negative CSF cultures throughout. In the Lzd group, 14 of the 15 patients with definite TBM showed CSF culture conversion from positive to negative, with culture results unknown in the remaining 1 patient; among the 17 patients with probable TBM, 15 remained culture-negative, while culture results were unknown in 2 patients. The CSF culture conversion rate was 100% (6/6) in the Czd group and 93.33% (14/15) in the Lzd group. Radiological assessment demonstrated lesion improvement in 13 of 16 patients (81.25%) in the Czd group, with 2 patients showing no significant change and 1 patient with unknown results. In the Lzd group, 26 of 32 patients (81.25%) showed improvement, 4 patients showed no significant change, and 2 patients had unknown results. Regarding changes in CSF parameters before and after 2 months of treatment (**Figure 2**), both groups demonstrated improvements across all measured CSF parameters. No statistically significant differences were observed between the two groups in the trends of any of the indicators: intracranial pressure (P = 0.830), CSF lactate (P = 0.662), CSF glucose (P = 0.452), CSF adenosine deaminase (ADA) (P = 0.917), lactate dehydrogenase (LDH) (P = 0.518), CSF chloride (P = 0.177), and CSF protein (P = 0.654). These findings indicate that Czd demonstrates favorable therapeutic efficacy in the treatment of tuberculous meningitis, which is consistent with previous research (Fan et al., 2025).

**Table 2:** Clinical details and outcomes of patients treated with Czd (n=16) or Lzd (n=32).

No.	Sex/Age	Resistance category	Mixed intracranial infection	TBM	BMRC	Clinical regimen	Dosage	AEs	Outcome			Comment
									Mtb	Symptoms	Radiology	
1	M/25	Drug-susceptible	–	Probable	II	H-R-E-Z-Lfx-Czd	Czd, 0.8g q12h	–	Negative	Improved	Unknown	Lzd-to-Czd (Lzd: 1 m), no Czd-related AEs
2	F/76	Drug-susceptible	–	Definite	II	H-R-Mfx-Czd	Czd, 0.4g q12h	–	Negative	Improved	Improved	Lzd-to-Czd (Lzd: 3 m), no Czd-related AEs
3	M/30	Drug-susceptible	–	Definite	II	Cs-Z-Mfx-Czd	Czd, 0.8g q12h	–	Negative	Improved	Unchanged	No Czd-related AEs
4	M/61	Drug-susceptible	–	Definite	II	H-Z-E-Czd	Czd, 0.8g q12h	–	Negative	Improved	Improved	Lzd-to-Czd (Lzd: 10 d), no Czd-related AEs
5	F/74	Drug-susceptible	–	Probable	II	H-Mfx-Cfz-Czd	Czd, 0.4g q12h	–	Negative	Improved	Improved	No Czd-related AEs
6	F/38	Drug-susceptible	–	Probable	II	H-R-E-Mfx-Czd	Czd, 0.8g q12h	–	Negative	Improved	Improved	Lzd-to-Czd (Lzd: 10 d), no Czd-related AEs
7	F/58	Drug-susceptible	–	Probable	II	CS-Lfx-Pto-Czd	Czd, 0.8g q12h	–	Negative	Improved	Improved	Lzd-to-Czd (Lzd: 8 m), no Czd-related AEs
8	F/26	Drug-susceptible	–	Probable	II	Am-Lfx-Czd	Czd, 0.8g q12h	–	Negative	Improved	Improved	Lzd-to-Czd (Lzd: 6 m), no Czd-related AEs
9	M/70	Isoniazid-resistance	–	Definite	III	R-Z-Mfx-Czd	Czd, 0.4g q12h	–	Negative	Improved	Improved	Lzd-to-Czd (Lzd: 15 d), no Czd-related AEs
10	F/62	Drug-susceptible	–	Probable	II	H-E-Lfx-Czd	Czd, 0.8g q12h	–	Negative	Improved	Improved	No Czd-related AEs
11	F/82	Drug-susceptible	–	Probable	I	H-E-Lfx-Czd	Czd, 0.8g q12h	–	Negative	Improved	Improved	No Czd-related AEs
12	M/45	Drug-susceptible	–	Probable	II	H-E-Lfx-Czd	Czd, 0.8g q12h	–	Negative	Improved	Improved	No Czd-related AEs
13	F/53	Drug-susceptible	–	Probable	I	H-E-Lfx-Czd	Czd, 0.8g q12h	–	Negative	Improved	Unchanged	No Czd-related AEs
14	M/32	Drug-susceptible	–	Definite	I	H-R-E-Lfx-Czd	Czd, 0.8g q12h	–	Negative	Improved	Improved	No Czd-related AEs
15	F/63	Drug-susceptible	–	Definite	II	H-Z-Cs-Czd	Czd, 0.4g q12h	–	Negative	Improved	Improved	Lzd-to-Czd (Lzd: 3.5 m), no Czd-related AEs
16	F/92	Drug-susceptible	–	Probable	II	H-Lfx-Czd	Czd, 0.8g q12h	–	Negative	Improved	Improved	Lzd-to-Czd (Lzd: 5 m), no Czd-related AEs
17	M/29	Multidrug-resistant	–	Definite	II	Lfx-Lzd-Cs-Z	Lzd, 0.6g qd	Anemia, Thrombocytopenia, Leukopenia	Negative	Improved	Improved	Dose interruptions of Lzd; Anemia correction therapy
18	F/28	Drug-susceptible	Neurosyphilis	Probable	II	H-R-Z-Mfx-Lzd	Lzd, 0.6g/0.3g qd	Vision decreased, Peripheral neuropathy	Negative	Improved	Improved	Dose reduction of Lzd
19	M/11	Multidrug-resistant	–	Definite	I	Lzd-Lfx-Cs-Z-Am	Lzd, 0.6g qd	Peripheral neuropathy, Hyperlactatemia	Negative	Improved	Improved	Hyperlactatemia correction therapy
20	F/68	Multidrug-resistant	–	Probable	II	Lzd-Cs-Cfz-Z-Am	Lzd, 0.6g/0.3g qd	Hyperlactatemia, Anemia	Negative	Improved	Improved	Dose reduction/interruptions of Lzd; Hyperlactatemia correction therapy
21	M/58	Drug-susceptible	–	Probable	I	H-R-Z-Lfx-Lzd	Lzd, 0.6g qd	Anemia	Unknown	Improved	Improved	–
22	M/51	Drug-susceptible	–	Probable	II	H-L-Z-Lfx-Lzd	Lzd, 0.6g/0.3g qd	Anemia, Thrombocytopenia, Leukopenia, Hyperlactatemia	Negative	Improved	Unchanged	Dose reduction/interruptions of Lzd
23	F/72	Drug-susceptible	–	Definite	II	H-R-E-Z-Lzd-Lfx	Lzd, 0.6g qd	Anemia	Unknown	Improved	Improved	Anemia correction therapy
24	M/19	Rifampicin-resistant	–	Probable	II	Lfx-Lzd-Lfx-Cs-Cfz-Am	Lzd, 0.6g qd	–	Negative	Improved	Improved	–
25	M/56	Drug-susceptible	–	Definite	I	Lzd-Cs-E-Lfx	Lzd, 0.6g/0.3g qd	Hyperlactatemia, Peripheral neuropathy	Negative	Improved	Unchanged	Dose reduction/interruptions of Lzd; Discontinued due to AEs
26	M/56	Drug-susceptible	Escherichia coli	Probable	II	Lzd-H-Mfx-Cs	Lzd, 0.6g/0.3g qd	Anemia, Thrombocytopenia, Peripheral neuropathy	Negative	Improved	Improved	Dose reduction of Lzd
27	F/39	Drug-susceptible	Viral meningitis	Probable	I	H-L-Z-Lzd-Mfx	Lzd, 0.6g qd	Anemia, Vision decreased	Negative	Improved	Improved	–
28	M/16	Drug-susceptible	–	Definite	I	H-R-Lfx-Lzd	Lzd, 0.6g qd	–	Negative	Improved	Unchanged	–
29	M/26	Drug-susceptible	–	Probable	II	H-R-Z-Lzd-Lfx-Am	Lzd, 0.6g qd	–	Negative	Improved	Improved	–
30	F/40	Multidrug-resistant	–	Probable	II	Mfx-Lzd-Z-Am	Lzd, 0.6g qd	Anemia, Hyperlactatemia, Peripheral neuropathy	Negative	Improved	Improved	Anemia correction therapy
31	M/16	Drug-susceptible	–	Definite	I	Lzd-Mfx-Am-H-R-Z	Lzd, 0.6g qd	–	Negative	Improved	Improved	–
32	F/30	Drug-susceptible	–	Probable	I	H-E-Lfx-Lzd	Lzd, 0.6g/0.3g qd	Anemia, Hyperlactatemia, Peripheral neuropathy	Negative	Improved	Improved	Dose interruptions of Lzd; Anemia correction therapy
33	M/67	Drug-susceptible	–	Definite	II	H-R-E-Z-Lzd	Lzd, 0.6g qd	Hyperlactatemia	Negative	Improved	Improved	–
34	M/73	Drug-susceptible	–	Definite	II	H-R-Z-Lfx-Lzd	Lzd, 0.6g qd	Hyperlactatemia	Negative	Improved	Unknown	–
35	F/55	Drug-susceptible	–	Probable	II	H-R-Z-Mfx-Lzd	Lzd, 0.6g qd	Hyperlactatemia, Anemia, Thrombocytopenia	Negative	Improved	Unchanged	Anemia and Thrombocytopenia correction therapy
36	M/64	Drug-susceptible	–	Probable	II	H-E-Lzd-Am-Cs	Lzd, 0.6g qd	Anemia, Hyperlactatemia	Negative	Improved	Improved	Anemia correction therapy; Discontinued due to AEs
37	M/36	Drug-susceptible	–	Definite	II	H-R-E-Z-Mfx-Lzd	Lzd, 0.6g/0.3g qd	Peripheral neuropathy	Negative	Improved	Improved	Dose reduction/interruptions of Lzd; Discontinued due to AEs
38	M/50	Isoniazid-resistance	–	Definite	II	R-E-Z-Lfx-Lzd	Lzd, 0.6g qd	–	Negative	Improved	Improved	–
39	M/81	Drug-susceptible	–	Probable	II	H-R-E-Lfx-Lzd	Lzd, 0.6g qd	Hyperlactatemia	Negative	Improved	Improved	–
40	F/36	Drug-susceptible	–	Probable	I	H-L-Am-Lfx-Lzd	Lzd, 0.6g/0.3g qd	–	Negative	Improved	Improved	–
41	M/68	Drug-susceptible	–	Definite	II	Am-Lfx-Lzd-Cs	Lzd, 0.6g/0.3g qd	Peripheral neuropathy, Vision decreased, Anemia	Negative	Improved	Improved	Dose reduction/interruptions of Lzd; Discontinued due to AEs
42	F/21	Drug-susceptible	–	Definite	II	H-R-Z-Lfx-Lzd	Lzd, 0.6g qd	–	Negative	Improved	Improved	–
43	M/33	Drug-susceptible	–	Definite	I	H-R-Z-Mfx-Lzd	Lzd, 0.6g qd	–	Negative	Improved	Unknown	–
44	M/59	Drug-susceptible	–	Probable	I	H-E-Lzd-Lfx	Lzd, 0.6g/0.3g qd	Peripheral neuropathy	Negative	Improved	Improved	Dose reduction/interruptions of Lzd; Discontinued due to AEs
45	F/40	Drug-susceptible	–	Probable	I	H-Lzd-Mfx-Cs	Lzd, 0.6g/0.3g qd	Peripheral neuropathy	Negative	Improved	Improved	Dose reduction/interruptions of Lzd; Discontinued due to AEs
46	F/29	Drug-susceptible	–	Definite	I	H-R-Mfx-Am-Lzd	Lzd, 0.6g qd	–	Negative	Improved	Improved	–
47	M/59	Drug-susceptible	–	Probable	II	H-R-Z-Lfx-Lzd	Lzd, 0.6g qd	Peripheral neuropathy	Negative	Improved	Improved	Discontinued due to AEs
48	M/61	Drug-susceptible	–	Definite	II	Lfx-Lzd-H-E	Lzd, 0.6g qd	–	Negative	Improved	Improved	–

Czd, contezolid; Lzd, linezolid; AEs, adverse events; d, days; m, months; q12h, every 12 hours; qd, once daily; H, isoniazid; R, rifampicin; E, ethambutol; Z, pyrazinamide; Lfx, levofloxacin; Mfx, moxifloxacin; Cfz, clofazimine; Cs, cycloserine; Pto, prothionamide; Am, amikacin; Lzd-to-Czd, switched from linezolid to contezolid.

To further clarify the efficacy of Czd in different clinical contexts, we performed a disaggregated subgroup analysis comparing the Czd-naive subgroup (n=7) and the Lzd-to-Czd switched subgroup (n=9). In the Czd-naive subgroup, all 7 patients (100%) achieved clinical symptom improvement. Among the patients with definite TBM, CSF Mtb culture conversion was achieved in all patients (100%), while the patients with probable TBM maintained negative cultures throughout. Radiological improvement was observed in 5 of 7 patients (71.43%), with 2 patients showing no significant change. In the Lzd-to-Czd switched subgroup, all 9 patients (100%) achieved clinical symptom improvement. Among the patients with definite TBM, CSF Mtb culture conversion was achieved in all patients (100%), while the patients with probable TBM maintained negative cultures throughout. Radiological improvement was observed in 8 of 9 patients (88.89%), with 1 patient showing unknown results. Regarding CSF parameters, longitudinal data were available for 11 patients (2 in the Czd-naive subgroup and 9 in the Lzd-to-Czd subgroup); the remaining 5 patients had normal baseline CSF parameters and did not undergo follow-up lumbar puncture. Notably, the Pre values in these data represent measurements taken at the time of Lzd discontinuation (immediately before Czd initiation), and the Post values represent measurements after approximately 2 months of Czd treatment. Both subgroups demonstrated consistent improvements across all measured CSF parameters following Czd treatment, including reductions in intracranial pressure, CSF protein, and lactate levels, alongside increases in CSF glucose and chloride levels (**Figure 2**). These findings suggest that Czd maintains favorable efficacy regardless of whether used as initial therapy or as salvage therapy after Lzd intolerance, though the small sample size precludes definitive statistical comparisons between the subgroups.

### 3.3 Safety evaluation

**Figure 3:**
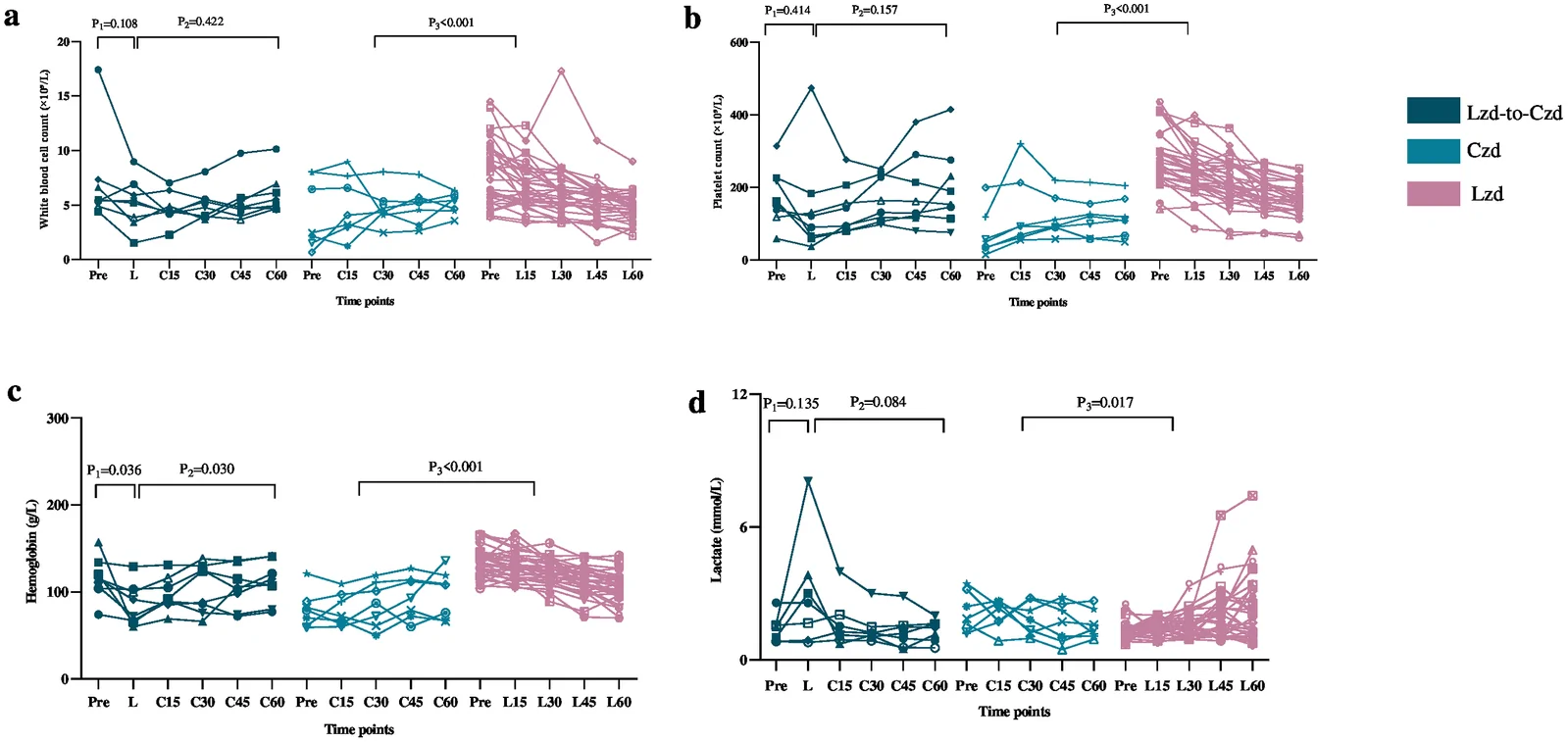
Individual trajectories of hematologic parameters and lactate in patients receiving Czd and Lzd containing regimens. Each panel shows individual patient trajectories for **(a)** white blood cell count, **(b)** platelet count, **(c)** hemoglobin, and **(d)** lactate levels. In the Lzd-to-Czd group (switch therapy), each line represents values at baseline (Pre), at Lzd discontinuation **(L)**, and after 15 days (C15), 30 days (C30), 45 days (C45), and 60 days (C60) of Czd treatment. In the Czd group (Czd-naive therapy), each line represents values at baseline (Pre) and after 15 days (C15), 30 days (C30), 45 days (C45), and 60 days (C60) of Czd treatment. In the Lzd group (Lzd-naive therapy), each line represents values at baseline (Pre) and after 15 days (L15), 30 days (L30), 45 days (L45), and 60 days (L60) of Lzd treatment.

In the safety evaluation, the Czd group was divided into the Czd-naive group and the Lzd-to-Czd group, the latter consisting of patients initially treated with Lzd who were subsequently switched to Czd. **Figure 3** illustrates the longitudinal trajectories of white blood cell, platelet, hemoglobin, and blood lactate levels over the 2-month treatment period across the three groups: Czd-naive group (n=7), Lzd-to-Czd group (n=9), and Lzd group (n=32). For the Lzd-to-Czd group, 8 patients were included in the analyses of white blood cells, platelets, and hemoglobin. One patient was excluded because hematological parameters remained stable after switching, and further monitoring was discontinued due to patient-related factors or clinical decision-making. For lactate analysis, 7 patients from the switch group were included, as two patients had normal initial lactate levels and were not followed up subsequently. Similarly, in the Lzd group, 30 patients were included in the lactate analysis, with two patients excluded for the same reason of normal initial values without further monitoring.

In the Lzd-to-Czd group (n=9), all patients were switched from Lzd to Czd due to intolerance to Lzd-related adverse reactions. The reasons for discontinuation included pancytopenia in 7 patients, elevated lactate in 1 patient, and visual blurring in 1 patient. Notably, after switching to Czd, all AEs completely resolved in every patient. In the within-group comparison of the Lzd-to-Czd group, hemoglobin showed a significant decline from baseline to Lzd discontinuation (P_1_=0.036) and a significant recovery after switching to Czd (P_2_=0.030). In the between-group comparison of the two treatment-naive groups, the P_3_ values for all four parameters were < 0.05, indicating that the Czd group exhibited stable trends across all parameters, whereas the Lzd group showed progressive decline or deterioration.

As shown in **Figure 3**, all 7 patients in the Czd-naive group presented with baseline cytopenias, which placed them at high risk for Lzd-related toxicity. Consequently, Czd was selected as the initial oxazolidinone agent from the outset. These patients tolerated Czd well throughout the entire treatment course, with no AEs reported and hematological parameters remaining stable relative to baseline.

In stark contrast, the Lzd group exhibited a substantial burden of AEs. Among the 32 patients treated with Lzd, 11 (34.38%) required dose reduction or temporary interruption due to AEs, and 7 (21.88%) permanently discontinued the drug. The spectrum of Lzd-related toxicities included anemia in 12 patients (37.5%), elevated lactate in 11 patients (34.4%), peripheral neuropathy in 11 patients (34.4%), thrombocytopenia in 4 patients (12.5%), visual blurring in 3 patients (9.4%), and leukopenia in 2 patients (6.3%). The high incidences of anemia and lactate elevation are consistent with the well-recognized mitochondrial toxicity of Lzd. Meanwhile, the elevated rate of peripheral neuropathy (34.4%) underscores the cumulative neurotoxic risk associated with prolonged oxazolidinone exposure. Importantly, 8 patients required specific interventions, including blood transfusions or platelet support, reflecting the clinical severity of these AEs and the substantial healthcare resource burden they impose, which further highlights a critical limitation of Lzd in the long-term management of TBM.

## 4 Discussion

In this real-world retrospective cohort study, we evaluated the clinical outcomes of Czd in patients with TBM, encompassing its role as salvage therapy in patients intolerant to Lzd and as initial treatment in those who avoided Lzd due to high-risk factors for AEs such as myelosuppression. Our findings demonstrated that although Lzd-related AEs were nearly universal, switching to Czd effectively reversed these events and restored hematological parameters toward baseline levels. Furthermore, in patients who received Czd as initial therapy, no significant changes in hematological parameters or lactate levels were observed, indicating a favorable safety profile. Comparative analysis of CSF parameters, CSF culture results, and radiological findings further demonstrated that Czd achieved comparable therapeutic efficacy to the Lzd group. Importantly, in the Lzd-to-Czd subgroup, CSF parameters were measured at Lzd discontinuation (Pre, immediately before Czd initiation) and after 2 months of Czd treatment (Post). The significant improvements observed from Pre to Post occurred after Czd therapy, indicating that the efficacy is attributable to Czd rather than a carry-over effect from prior Lzd treatment. Even in the two patients with prolonged Lzd exposure (≥5 months), the Pre-to-Post improvements only suggest that Czd further improved CSF parameters after Lzd discontinuation, rather than reflecting a carry-over effect of Lzd.

The safety evaluation demonstrates that Czd possesses a favorable and distinctively superior safety profile compared with Lzd in the treatment of TBM. However, for white blood cells, platelets, and lactate, neither P_1_ nor P_2_ reached statistical significance (all P > 0.05) in the Lzd-to-Czd group, which may be attributable to the following factors. First, the small sample size of the Lzd-to-Czd group (n = 9) limited statistical power to detect moderate effect sizes. Second, the duration of prior Lzd exposure in the switch group varied considerably (range: 10 days to 8 months; median: 3 months), with 3 patients exposed for ≤ 15 days, 4 patients for 1 to 3.5 months, and 3 patients for ≥ 5 months. Patients with shorter exposure may not have accumulated sufficient drug-related toxicity, while those with longer exposure had already developed substantial toxicity. This heterogeneity increased within-group variability and attenuated the statistical significance of P_1_ and P_2_.

From a clinical management perspective, Czd should be considered the preferred oxazolidinone agent for TBM patients with baseline cytopenias, as it provides effective therapy without exacerbating pre-existing hematological impairments. For patients already on Lzd therapy, hemoglobin should be closely monitored as the earliest and most reliable indicator of bone marrow suppression; a significant decline should prompt timely consideration of switching to Czd. Furthermore, the high incidence of Lzd-related peripheral neuropathy underscores the critical importance of early recognition and proactive avoidance. This safety evaluation demonstrates that Czd offers a clinically meaningful safety advantage over Lzd in the treatment of TBM, particularly in patients with baseline cytopenias or those who have developed Lzd-related toxicities. The excellent tolerability observed in the Czd group further supports Czd as a valuable therapy for Lzd-intolerant patients or those with pre-existing cytopenias precluding Lzd use.

Our team recently published a retrospective study on Czd for the treatment of complex TB (Lv et al., 2026). During the initial case screening process of the present study, we identified a similar issue: although Czd demonstrates favorable efficacy and safety, its high cost poses a substantial obstacle (Zhang et al., 2022). We initially identified 39 patients diagnosed with definite or probable TBM between March 2022 and March 2026 who received Czd-containing regimens, yet only 16 patients completed treatment for more than 2 months. Among the remaining 23 patients who discontinued treatment within 2 months, 18 patients discontinued due to financial burden. Future pharmacoeconomic studies are warranted to comprehensively evaluate the cost-effectiveness of Czd versus Lzd in TB treatment, taking into account both direct drug costs and indirect expenses associated with AE management and treatment prolongation.

The statistically significant baseline differences between groups, including fever (87.5% vs. 46.88%, P = 0.007) and consciousness disturbance (62.5% vs. 21.88%, P = 0.006), reflect the clinical reality that patients with more severe presentations were more likely to receive Czd. This confounding by indication is an inherent limitation of non-randomized real-world studies. The low nausea/vomiting rate in the Czd group may partly reflect symptom reporting bias, as 62.5% of these patients had consciousness disturbance and could not reliably report subjective symptoms. Importantly, the core safety findings—toxicity reversal in the Lzd-to-Czd group (assessed via within-patient paired comparisons) and the absence of AEs in the Czd-naive group—are unaffected by these baseline imbalances. The efficacy comparisons, showing no significant differences in CSF parameter trends, are descriptive and do not rely on strict baseline comparability.

Several limitations should be acknowledged, including the small sample size of the Czd group and the retrospective design, which may introduce potential selection bias. Current clinical evidence on Czd for the treatment of TBM is predominantly derived from case reports, and future prospective studies with larger sample sizes are warranted to further validate its impact on CSF parameters and its clinical value in TBM therapy. Additionally, the 2-month follow-up period in this study is relatively short for TBM, which typically requires 6 to 12 months of treatment. This limits our ability to assess long-term outcomes such as functional recovery, neurological sequelae, and disease relapse. Longer follow-up is needed to confirm the durability of the favorable responses observed with Czd. Furthermore, the exclusion of patients who discontinued Czd treatment within 2 months—largely due to financial constraints—may introduce attrition/survivorship bias. Patients who were able to complete the 2-month treatment course may have had better socioeconomic support, more comprehensive overall care, or potentially less severe disease, which could limit the generalizability of our findings to the broader Czd-treated TBM population, particularly those facing economic barriers to treatment adherence. Despite these limitations, our study provides valuable real-world evidence demonstrating that Czd has a favorable safety and efficacy profile in the treatment of TBM. These findings support the use of Czd in TBM therapy, particularly as an alternative for patients intolerant to Lzd.

## 5 Conclusion

In conclusion, our study demonstrates that Czd plays a beneficial role in the management of TBM. It serves as an effective salvage therapy for patients intolerant to Lzd, with favorable safety and sustained efficacy. The stable hematologic and metabolic profile observed in selected high-risk patients receiving Czd as initial therapy supports its use as a salvage or alternative option in those who cannot tolerate Lzd. Although our findings provide valuable real-world evidence, further prospective studies and randomized controlled trials with larger sample sizes are warranted to confirm these observations and refine treatment strategies for TBM.

## Data Availability

The original contributions presented in the study are included in the article/supplementary material. Further inquiries can be directed to the corresponding author.
